# GNSS Single Frequency, Single Epoch Reliable Attitude Determination Method with Baseline Vector Constraint

**DOI:** 10.3390/s151229774

**Published:** 2015-12-02

**Authors:** Ang Gong, Xiubin Zhao, Chunlei Pang, Rong Duan, Yong Wang

**Affiliations:** Information and Navigation School, Air Force Engineering University, No.1 FengHao Road, LianHu District , Xi’an 710077, China; sweeteve@sina.cn(A.G); zhaoxiubin926@163.com (X.Z.); duanrong0919@126.com (R.D.); wangyongnav@163.com (Y.W.)

**Keywords:** GNSS, baseline vector constraint, ambiguity, attitude determination

## Abstract

For Global Navigation Satellite System (GNSS) single frequency, single epoch attitude determination, this paper proposes a new reliable method with baseline vector constraint. First, prior knowledge of baseline length, heading, and pitch obtained from other navigation equipment or sensors are used to reconstruct objective function rigorously. Then, searching strategy is improved. It substitutes gradually Enlarged ellipsoidal search space for non-ellipsoidal search space to ensure correct ambiguity candidates are within it and make the searching process directly be carried out by least squares ambiguity decorrelation algorithm (LAMBDA) method. For all vector candidates, some ones are further eliminated by derived approximate inequality, which accelerates the searching process. Experimental results show that compared to traditional method with only baseline length constraint, this new method can utilize *a priori* baseline three-dimensional knowledge to fix ambiguity reliably and achieve a high success rate. Experimental tests also verify it is not very sensitive to baseline vector error and can perform robustly when angular error is not great.

## 1. Introduction

GNSS double-differenced carrier phase observables can be used for high precision attitude determination, and the key is integer ambiguity resolution. Many methods are proposed and more recent ones make use of the LAMBDA method, see Furuno (2003) [1], Monikes (2005) [2], Kuylen (2006) [3], Hauschild (2008) [4], Wang (2009) [5], Chen (2013) [6], and Landry R2015 [7].

Standard LAMBDA method can be only used for unconstrained and/or linearly constrained GNSS models, but baseline constraint is nonlinear. So, most of them make use of this additional information by optimizing searching space and checking whether or not the candidate ambiguities satisfy the given baseline. Although this kind of process improves the performance of ambiguity resolution, they are still unable to reach a very high reliability, as prior information is not fully integrated in the ambiguity resolution process. Especially when used for single frequency, single epoch attitude determination success rate of ambiguity fixing decreases dramatically [8,9].

For single epoch attitude determination, Chansik Park and Teunissen propose their own nonlinear constrained integer least square method and relevant searching strategy [10,11]. Both methods integrate nonlinear constraints into ambiguity objective function, the former is based on LAMBDA ellipsoidal search space, and the latter is based on a more rigorous but complex non-ellipsoidal search space. Although they improve the success rate to a high degree compared with unconstrained issues, it is not high enough for reliable application. As the same with most methods before, they only concern the baseline length as prior information. Concerning baseline length as well as angular information, Henkel extends baseline constraints to three-dimensions, makes use of baseline length and orientation for ambiguity maximum posterior estimation, and an estimator is designed [12,13]. Although it is very innovative in utilizing length and angular information of baseline vector, prior information is not fully integrated into ambiguity objective function, and it is used for application with multi-epoch and dual-frequency. In recent years, other research on GNSS attitude determination methods without combination mainly focuses on misaligned baselines [14], instantaneous ambiguity resolution and attitude determination [15], and medium-length baselines and multi-frequency single epoch attitude determination [16,17]. However these methods do not accommodate whole prior baseline vector information into constraint and only take baseline length into consideration.

In this contribution, we emphasize the real-time requirement of aircraft attitude determination and propose a reliable single frequency, single epoch attitude determination method with baseline vector constraints. This method utilizes baseline rough three-dimensional prior information obtained by other airborne equipment such as compass and inertial navigation system. The contribution is organized as follows: First, we reconstruct ambiguity objective function with baseline length and angular information. Then, constrained integer least square resolution is proposed based on the function above, relevant ambiguity searching strategy is proposed too. Finally, we report the static experiment and dynamic test and verify, both indicate the good performance of our proposed new method, with success rates achieving 100% in the model experiment.

## 2. Baseline Vector Constrained Model

GNSS double differenced linearized observation equation:

(1) y=Aa+Bb+e

where y is the data vector of order m, consists of carrier phase and code observations and a is integer ambiguity of order n, b is unknown parameter vector of order p consists of three-dimensional baseline coordinates and atmosphere delay parameter. In the GNSS attitude model, the baseline is generally very short in order to eliminate atmosphere delay and meet the requirement of real-time computation [18], thus p=3.

Two antennas are used for attitude determination, one is placed in the middle of the carrier body central axis, located at $\left[\begin{array}{ccc} 0 & 0 & 0 \end{array}\right]$, and the other one is located at $\left[\begin{array}{ccc} l & 0 & 0 \end{array}\right]$. Heading and pitch are derived by baseline vector according to following equation:

(2) $$\theta =\arctan\frac{b_{2}}{b_{1}}\;\beta =\arctan\frac{b_{3}}{\sqrt{b_{1}^{2}+b_{2}^{2}}}$$

where $b_{1},b_{2},b_{3}$ are the three coordinates of baseline vector. In order to reach a very high precision, integer ambiguity must be correctly resolved. Ambiguity integer least square solution without constraint is given as [19]:

(3) $${\overset{⌣}{a}}_{ILS}=\arg\underset{a\in Z^{n}}{\min}{\left\|\hat{a}-a\right\|}_{Q_{\hat{a}\hat{a}}}^{2}$$

where $\hat{a}$ is float solution, $Q_{\hat{a}\hat{a}}$ is variance matrix. As no prior information exists, there is any baseline related items in objective function.

When baseline vector prior information with length, heading, and pitch is taken into consideration, one can write Equation (3) as [20]:

(4) $$\begin{array}{l} {\overset{⌣}{a}}_{CILS}=\arg\underset{a\in Z^{n}}{\min}F(a)\\ \;=\arg\;\underset{a\in Z^{n}}{\min}\left\{{||\hat{a}-a||}_{Q_{\hat{a}\hat{a}}}^{2}+\underset{b\in R^{3}}{\min}H(a,b)\right\} \end{array}$$

(5) $$H(a,b)={||\hat{b}(a)-b||}_{Q_{\hat{b}(a)\hat{b}(a)}}^{2}+\frac{{\left(\theta -\bar{\theta }\right)}^{2}}{\delta_{\theta }^{2}}+\frac{{\left(\beta -\bar{\beta }\right)}^{2}}{\delta_{\beta }^{2}}+\frac{{\left(l-\bar{l}\right)}^{2}}{\delta_{l}^{2}}$$

(6) $$\theta =\arctan\frac{b{\left(a\right)}_{2}}{b{\left(a\right)}_{1}}\;\beta =\arctan\frac{b{\left(a\right)}_{3}}{\sqrt{b{\left(a\right)}_{1}^{2}+b{\left(a\right)}_{2}^{2}}}\;l=||b(a)||$$

The objective function is very different from any other methods before as it integrates angular information into the function, see Equation (5), where θ, β, l are the components of baseline vector in sphere coordinate system, namely heading, pitch, and length. $\bar{\theta },\;\bar{\beta },\;\bar{l}$ stand for true value, in the computation we use prior measurement as substitution. $\delta_{\theta }^{2},\delta_{\beta }^{2},\delta_{l}^{2}$ is the variance of θ,β,l, respectively. Generally, l can be measured very precisely, and $\delta_{l}^{2}$ is very small. θ and β are measured by other airborne navigation systems and sensors with rough precision and bigger variance.

## 3. Baseline Vector Constrained Ambiguity Searching Algorithm

### 3.1 Search Space Determination

Assuming a known, then the minimizer of H(a,b) in Equation (5) is given as:

(7) $$\overset{⌣}{b}(a)=\underset{b\in R^{p}}{\arg\;\min}H(a,b)$$

This nonlinear LS solution is calculated iteratively as follows:

(8) $$\overset{⌣}{b}{\left(a\right)}^{\left(k+1\right)}=\overset{⌣}{b}{\left(a\right)}^{\left(k+1\right)}-\nabla^{2}H{\left(a,\overset{⌣}{b}{\left(a\right)}^{\left(k\right)}\right)}^{-1}\cdot \nabla H\left(a,\overset{⌣}{b}{\left(a\right)}^{\left(k\right)}\right)$$

where $\nabla H(\cdot )=\left[\begin{array}{c} \frac{\partial H}{\partial b_{1}}\\ \frac{\partial H}{\partial b_{2}}\\ \frac{\partial H}{\partial b_{3}} \end{array}\right]\;\nabla^{2}H(\cdot )=\left[\begin{array}{ccc} \frac{\partial^{2}H}{\partial^{2}{b_{1}}^{2}} & \frac{\partial^{2}H}{\partial b_{1}\partial b_{2}} & \frac{\partial^{2}H}{\partial b_{1}\partial b_{3}}\\ \frac{\partial^{2}H}{\partial b_{2}\partial b_{1}} & \frac{\partial^{2}H}{\partial^{2}{b_{2}}^{2}} & \frac{\partial^{2}H}{\partial b_{2}\partial b_{3}}\\ \frac{\partial^{2}H}{\partial b_{3}\partial b_{1}} & \frac{\partial^{2}H}{\partial b_{3}\partial b_{2}} & \frac{\partial^{2}H}{\partial^{2}{b_{3}}^{2}} \end{array}\right]$

It can be initialized by $\hat{b}(a)={\left(B^{T}Q_{yy}^{-1}B\right)}^{-1}B^{T}Q_{yy}^{-1}(y-Aa)$. Combining Equations (7) and (4) then gives:

(9) $$F(a)={||\hat{a}-a||}_{Q_{\hat{a}\hat{a}}}^{2}+H\left(a,\overset{⌣}{b}(a)\right)$$

${\overset{⌣}{a}}_{CILS}$ is computed by search, the searching space $\Omega \subset Z^{n}$ is defined as:

(10) $$\Omega_{\left(F\right)}=\left\{a\in Z^{n}|F(a)\leq \chi^{2}\right\}$$

Different from standard LAMBDA method, F(a) is not a quadratic form of a, and it cannot be formulated as weighted square sum. Thus, it is difficult to determine the ambiguities search scope sequentially as standard LAMBDA method does, but from Equation (9) we know that for a given $\chi^{2}$, $F(a)\leq \chi^{2}$, there must exist ${||\hat{a}-a||}_{Q_{\hat{a}\hat{a}}}^{2}\leq \chi^{2}$, and $\Omega_{\left(E\right)}=\left\{a\in Z^{n}|{\left\|\hat{a}-a\right\|}_{Q_{\hat{a}\hat{a}}}^{2}\leq \chi^{2}\right\}$ is just an ellipsoidal search space. So, for a given value $\chi^{2}$, $\Omega_{\left(F\right)}\subset \Omega_{\left(E\right)}$, enumerates all the candidate vectors in $\Omega_{\left(E\right)}$ then one can find the minimizer of F(a). Although it is somehow exhaustive of substituting $\Omega_{\left(E\right)}$ for $\Omega_{\left(F\right)}$ during the search process, it is feasible and applicable. The only problem is that the search space is possibly expanded to a much bigger one than the actual non-ellipsoidal one, due to the following reason.

When using single frequency, single epoch data, in Equation (1) $A=\left[\lambda I_{n},0\right],\;B={\left[G^{T},G^{T}\right]}^{T}$. Let $\sigma_{\phi }^{2}Q$ and $\sigma_{\rho }^{2}Q$ stands for phase and code measurement variance matrix respectively [11], then ambiguity variance matrix and conditional baseline variance matrix are as follow respectively:

(11) $$\begin{array}{l} Q_{\hat{a}\hat{a}}={\left({\bar{A}}^{T}Q_{yy}^{-1}\bar{A}\right)}^{-1}=\frac{\sigma_{\rho }^{2}}{\lambda^{2}}\left(\frac{\sigma_{\rho }^{2}}{\lambda^{2}}Q+G(G^{T}Q^{-1}G{)}^{-1}G^{T}\right)\\ \;\approx \frac{\sigma_{\rho }^{2}}{\lambda^{2}}G(G^{T}Q^{-1}G{)}^{-1}G^{T} \end{array}$$

(12) $$\begin{array}{l} Q_{\hat{b}(a)\hat{b}(a)}={\left(B^{T}Q_{yy}^{-1}B\right)}^{-1}=\frac{\sigma_{\phi }^{2}}{1+\sigma_{\phi }^{2}/\sigma_{\rho }^{2}}{\left(G^{T}Q^{-1}G\right)}^{-1}\\ \;\approx \sigma_{\phi }^{2}{\left(G^{T}Q^{-1}G\right)}^{-1} \end{array}$$

Generally, $\sigma_{\phi }^{2}\ll \sigma_{\rho }^{2}$. Thus, for most ambiguity candidates a, the first item ${\left\|\hat{a}-a\right\|}_{Q_{\hat{a}\hat{a}}}^{2}$ is much smaller than ${\left\|\hat{b}(a)-b\right\|}_{Q_{\hat{b}(a)\hat{b}(a)}}^{2}$ in Equation (9), *i.e.*, ${\left\|\hat{a}-a\right\|}_{Q_{\hat{a}\hat{a}}}^{2}\ll F(a)$. If the size of search space $\Omega_{E}(\chi^{2})$ is set by $\chi^{2}=F(a)$, it will be expanded too much and contains too many candidate vectors, which makes the search tend to become inefficient or interrupted.

**Figure 1:**
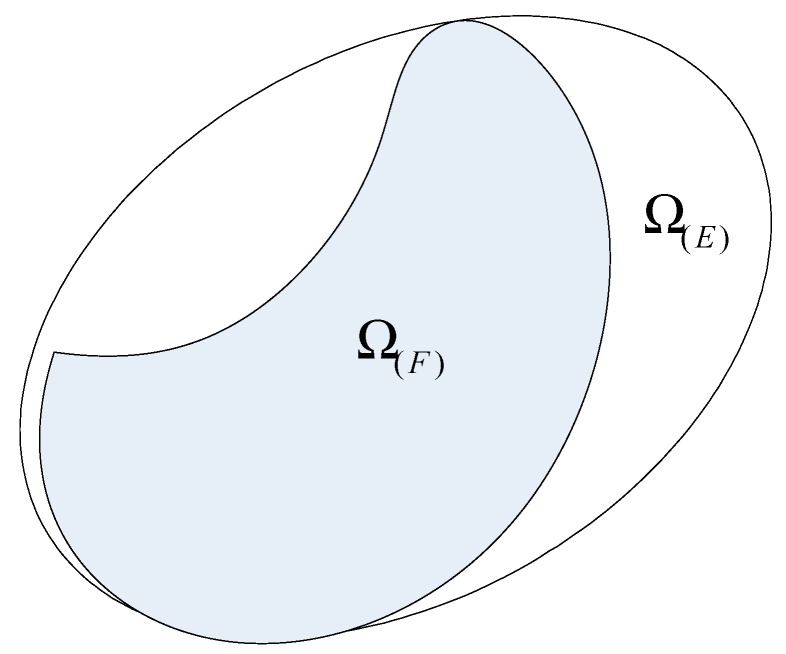
Search spaces of encompassment: $\Omega_{\left(F\right)}$ is the non-ellipsoidal search space with constraint, $\Omega_{\left(E\right)}$ is the ellipsoidal search space without constraint.

In order to avoid a too large a search space, we can use ${\left\|\hat{a}-a\right\|}_{Q_{\hat{a}\hat{a}}}^{2}$ to set a smaller $\chi_{E}^{2}$ at first as it does in [10], but this operation probably makes it too small to encompass $\Omega_{\left(F\right)}$. So, the best way is to find a proper $\Omega_{\left(E\right)}$ that encompass $\Omega_{\left(F\right)}$ and is as small as possible as Figure 1 shows.

In this contribution, we first set a smaller $\chi_{1}^{2}$ as initial value. If there are no candidates in the search space, then $\chi^{2}$ increases. Repeat this operation until the increased search space contains at least one candidate. Although this operation is simple, it can avoid exhaustive searching efficiently. As correct ambiguity can minimize F(a), we can set $\chi_{1}^{2}$ by $\chi_{1}^{2}={\left\|\hat{a}-a_{ILS}\right\|}_{Q_{\hat{a}\hat{a}}}^{2}$ with the solution of standard LAMBDA method.

### 3.2 Modified Search Strategy

One needs to enumerate all the candidate vectors in $\Omega_{\left(E\right)}$ and calculate the corresponding $\overset{⌣}{b}(a)$ to get $\underset{b\in R^{3}}{\min}H(a,b)$, *i.e.*, $H\left(a,\overset{⌣}{b}(a)\right)$. From Equation (8), it is nonlinear least square computation and complex. In order to accelerate the search process, one must reduce the calculation times of $\underset{b\in R^{3}}{\min}H(a,b)$ as many as possible.

For a given $\chi^{2}$ that guarantees the search space $\Omega_{\left(E\right)}$ is nonempty, there is at least one candidate vector $a_{i}$ in search space that satisfies the following inequality:

(13) $$F(a_{i})={\left\|\hat{a}-a_{i}\right\|}_{Q_{\hat{a}\hat{a}}}^{2}+\underset{b\in R^{3}}{\min}H\left(a,b(a_{i})\right)\leq \chi^{2},\;i.e.,\;\underset{b\in R^{3}}{\min}H\left(a,b(a_{i})\right)\leq \chi^{2}-{\left\|\hat{a}-a_{i}\right\|}_{Q_{\hat{a}\hat{a}}}^{2}$$

This inequality is used to check whether $\chi^{2}$ is of proper value that guarantee the search space is nonempty. And when $\chi^{2}$ is of proper value, it is further used to check whether other candidate vectors in $\Omega_{\left(E\right)}$ is contained in $\Omega_{\left(F\right)}$ as well. Only if $\underset{b\in R^{3}}{\min}H\left(a,b(a)\right)\leq \chi^{2}-{\left\|\hat{a}-a\right\|}_{Q_{\hat{a}\hat{a}}}^{2}$, then could the corresponding a probably be the correct solution of integer ambiguity. As it is very complex to calculate $\underset{b\in R^{3}}{\min}H(a,b)$, we can compute a tight or an approximately tight lower bound of $\underset{b\in R^{3}}{\min}H(a,b)$ instead of $\underset{b\in R^{3}}{\min}H(a,b)$ itself to utilize inequality Equation (13). The detail of this process is as follows:

For simplifying, we only concern the baseline length when compute the lower bound, *i.e.*, l=‖b(a)‖. Then Equation (5) is rewritten as:

(14) $$H^{\prime }(a,b)={\left\|\hat{b}(a)-b\right\|}_{Q_{\hat{b}(a)\hat{b}(a)}}^{2}+\frac{{\left(\left\|b\right\|-l\right)}^{2}}{\delta_{l}^{2}}$$

Assuming $Q_{\hat{b}(a)\hat{b}(a)}=\frac{1}{\lambda }I_{3}$ is a diagonal matrix, then the contour plane of ${\left\|\hat{b}(a)-b\right\|}_{Q_{\hat{b}(a)\hat{b}(a)}}^{2}$ is a sphere with the center at $\hat{b}(a)$, $\frac{{\left(\left\|b\right\|-l\right)}^{2}}{\delta_{l}^{2}}$ is also a sphere with the center at origin. So, the minimizer of $H^{\prime }(a,b)$, *i.e.*, ${\overset{⌣}{b}}^{\prime }(a)$ must be between $\hat{b}(a)$ and the origin. Let ${\overset{⌣}{b}}^{\prime }(a)=\mu \hat{b}(a)/\left\|\hat{b}(a)\right\|$, $0\leq \mu \leq \left\|\hat{b}(a)\right\|$, plugging this into Equation (14), then we get the value $\hat{\mu }=\frac{l+\sigma_{l}^{2}\lambda \left\|\hat{b}(a)\right\|}{1+\sigma_{l}^{2}\lambda }$ that minimizes $H^{\prime }(a,b)$. It is actually the weighted mean value of $\left\|\hat{b}(a)\right\|$ and l, then we get a proximate value of $\overset{⌣}{b}(a)$, that is:

(15) $${\overset{⌣}{b}}^{\prime }(a)=\arg\;\underset{b\in {\mathbb{R}}^{p}}{\min}H^{\prime }(a,b)=\frac{l+\sigma_{l}^{2}\lambda \left\|\hat{b}(a)\right\|}{1+\sigma_{l}^{2}\lambda }\cdot \frac{\hat{b}(a)}{\left\|\hat{b}(a)\right\|}\;\hat{b}(a)\neq 0$$

Turn it back to Equation (14):

(16) $$\underset{b\in {\mathbb{R}}^{p}}{\min}H^{\prime }(a,b)=\frac{\lambda {\left(l-\left\|\hat{b}(a)\right\|\right)}^{2}}{1+\sigma_{l}^{2}\lambda }$$

If $\hat{b}(a)=0$, $\overset{⌣}{b^{\prime }}(a)$ has no solution, minimized $H^{\prime }(a,b)$ is still $\frac{\lambda {\left(l-\left\|\hat{b}(a)\right\|\right)}^{2}}{1+\sigma_{l}^{2}\lambda }$.

Generally, $Q_{\hat{b}(a)\hat{b}(a)}$ is not a diagonal matrix, but the following inequality holds:

(17) $${\left\|\hat{b}(a)-b\right\|}_{\frac{1}{\lambda_{\min}}I_{p}}^{2}\leq {\left\|\hat{b}(a)-b\right\|}_{Q_{\hat{b}(a)\hat{b}(a)}}^{2}\leq {\left\|\hat{b}(a)-b\right\|}_{\frac{1}{\lambda_{\max}}I_{p}}^{2}$$

where, $\lambda_{\min}$ denotes the minimum eigenvalue of $Q_{\hat{b}(a)\hat{b}(a)}$. $\lambda_{\max}$ denotes the maximum. Then we get:

(18) $$\frac{\lambda_{\min}{\left(l-\left\|\hat{b}(a)\right\|\right)}^{2}}{1+\sigma_{l}^{2}\lambda_{\min}}\leq \min\left\{{\left\|\hat{b}(a)-b\right\|}_{Q_{\hat{b}(a)\hat{b}(a)}}^{2}+\frac{{\left(\left\|b\right\|-l\right)}^{2}}{\delta_{l}^{2}}\right\}\leq \frac{\lambda_{\max}{\left(l-\left\|\hat{b}(a)\right\|\right)}^{2}}{1+\sigma_{l}^{2}\lambda_{\max}}$$

(19) $$\frac{\lambda_{\min}{\left(l-\left\|\hat{b}(a)\right\|\right)}^{2}}{1+\sigma_{l}^{2}\lambda_{\min}}\leq \min H^{\prime }(a,b)<\min H(a,b)<\chi^{2}-{\left\|\hat{a}-a\right\|}_{Q_{\hat{a}\hat{a}}}^{2}$$

Utilize formula $\frac{\lambda_{\min}{\left(l-\left\|\hat{b}(a)\right\|\right)}^{2}}{1+\sigma_{l}^{2}\lambda_{\min}}<\chi^{2}-{\left\|\hat{a}-a\right\|}_{Q_{\hat{a}\hat{a}}}^{2}$ as checking inequality. Eliminate all the vectors that do not satisfy the inequality before calculating $\underset{b\in R^{3}}{\min}H(a,b)$ so as to make search more efficient.

Although $\min H^{\prime }(a,b)<\min H(a,b)$ is not a tight inequality, the two items are actually very approximate as baseline length is much more accurately measured than angle. So, ignoring angular information seldom adds candidate vectors. For computation, it is much easier to calculate $\min H^{\prime }(a,b)$ than minH(a,b).

The steps for computing $\overset{⌣}{a}$ are then as follows:

Step 1: With float solution $\hat{a},\hat{b}$ and relevant variance and covariance matrixes.

1. Compute the minimum eigenvalue of $Q_{\hat{b}(a)}=Q_{\hat{b}\hat{b}}-Q_{\hat{b}\hat{a}}Q_{\hat{a}\hat{a}}^{-1}Q_{\hat{a}\hat{b}}$;
2. Compute $a_{ILS}$ with unconstrained LAMBDA;
3. Initialize $\chi^{2}$ by $\chi_{ILS}^{2}={\left\|\hat{a}-a_{ILS}\right\|}_{Q_{\hat{a}\hat{a}}}^{2}$.

Step 2: For all the candidate vectors a that satisfy ${\left\|\hat{a}-a\right\|}_{Q_{\hat{a}\hat{a}}}^{2}\leq \chi^{2}$.

1. Compute $\hat{b}(a)=\hat{b}-Q_{\hat{b}\hat{a}}Q_{\hat{a}\hat{a}}^{-1}(\hat{a}-a)$;
2. If $\frac{\lambda_{\min}{\left(l-\left\|\hat{b}(a)\right\|\right)}^{2}}{1+\sigma_{l}^{2}\lambda_{\min}}<\chi^{2}-{\left\|\hat{a}-a\right\|}_{Q_{\hat{a}\hat{a}}}^{2}$, calculate $\overset{⌣}{b}(a)$ according to Equation (8), and then calculate F(a).

Step 3: If the search space is empty *i.e.*, no a satisfies the above inequality, expand the search space as $\chi^{2}=\chi^{2}+\chi_{ILS}^{2}$, and then repeat step2 till Ω is nonempty. Select one a that returns the smallest value for F(a) as fixed solution.

As long as the ambiguity is fixed, b=b(a) is the final high precise baseline solution to determine the attitude.

## 4. Test Verification and Analyses

In order to test the performance of proposed method, static and dynamic experiments are carried out on 23 April and 2 May 2015 respectively in Xi’an, China. With a 10° cut off elevation angle, seven and eight satellites are tracked. PDOP value is between about 1.5 and 2.1 during the static as well as dynamic experiments, which means good measuring environment.

### 4.1. Static Experiment 

In the static experiment, two antennas of GPS-702-GG are located at both ends and connected to two receivers of NovAtel OEM628 (NovAtel Inc, Calgary, AL. Canada), which can provide measurement precision about L1 carrier phase 2 mm (1σ) and C/A code measurement 20 cm (1σ). Carrier phase data of frequency L1 and C/A code data are both collected. Experiments are carried out two times with different baselines. In the first experiment, the baseline is of length 3.145 m, heading 25.025°, pitch 11.208°. In the second experiment, the baseline is of length 8.343 m, heading 49.402°, pitch −16.137°. The baseline length is measured by millimeter ruler with a standard deviation 0.5 mm. Angular prior information is obtained by the Inertial Navigation System coarse alignment, a type of Novatel SPAN-CPT(Synchronized Position Attitude Navigation-Compact, Portable, and Tightly Coupled，NovAtel Inc , Calgary, AL, Canada), and the standard deviation is heading 0.8° and pitch 0.6°.

2156 and 2013 epochs are logged respectively during the two experiments. Resolve the integer ambiguity with one epoch and another. Multi-epoch solution is regarded as true ambiguity. Compare the proposed method in this paper with BC-LAMBDA (BC stands for Baseline Constraint) method in [10] and BC-LAMBDA of rigorous non-ellipsoidal search strategy is proposed in [11]. The success rates and average computation time of each method are as shown in Table 1.

Both methods in [10,11] only concern baseline length constraint. As it shown in Table 1, method in [11] performs better, achieving a higher success rate, but it still gets ambiguity resolution wrong at over 10 epochs. Different from the tow methods above, this paper’s new method concerns not only baseline length but also angular constraint, thus to strengthen the model of GNSS attitude determination and achieve a success rate of 100% in two experiments of different baselines.

The whole time that was required for resolving integer ambiguity at every epoch was measured using Matlab. Then we divided the whole computation time by number of epochs to obtain the average computation time per epoch. The PC is of Pentium(R) Dual-Core CPU E5500+2.80 GHz, 2.79 GHz and 1.96 G RAM.

**Table 1:** Single frequency, single epoch success rate and average computation time comparison.

	Baseline Length/m	Epochs	Success Epochs	Success Rate/%	Average Computation Time/s
method in [10]	3.14	2156	2132	98.6	0.065
	8.34	2013	1957	96.7	0.093
method in [11]	3.14	2156	2142	99.4	0.084
	8.34	2013	1999	99.3	0.123
This new method	3.14	2156	2156	100	0.079
	8.34	2013	2013	100	0.116

As is shown in Table 1, this paper new method is a little slower than methods in [10,11], it is still very fast and very close to reference methods. The average computation time is much shorter than 1 s (1 epoch). Compared to method in [11], it performs better than ambiguity resolution, and the computation time does not increase because of the modified search strategy. With single epoch measurement, short computation time, and a very high ambiguity fix success rate, this new method can be expected for airborne application reliably.

**Figure 2:**
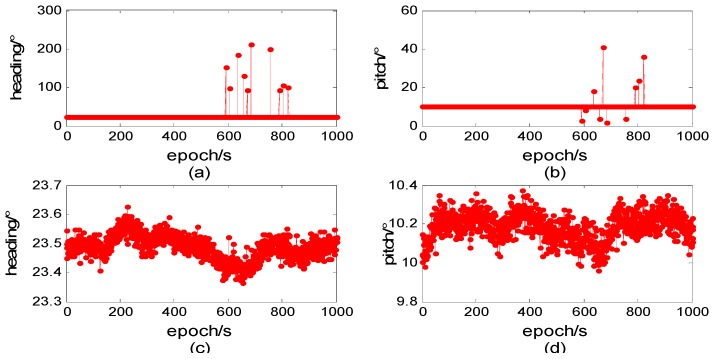
Attitude determination comparison. (**a**,**b**) are the results of method in [11]; (**c**) and (**d**) are results of new method.

In the first experiment, the attitude of baseline is determined with the first 1000 epochs, computing one by one. This paper’s proposed method is compared with method in [11], and the result is shown in Figure 2.

Figure 2 shows that the solution of heading and pitch are probably far from true value and shake severely at a few epochs when using method in [11]. This is actually due to the wrong fixed ambiguity. Although the baseline length holds still close to the true value, the heading and pitch deviated. When using baseline vector constraint of three-dimensions, we integrate heading and pitch constraint information into objective function. If the heading or pitch deviates from prior information to some extent, objective function increases dramatically, the relevant ambiguity candidate will not be chosen as fixed right one. This is why this paper proposed method performs better and achieves a higher success rate. Figure 2 also shows that after integer ambiguity is correctly fixed, the result of attitude determination in the experiment is heading 23.5°, about 1.5° deviation from prior information; pitch 10.2°, about 1.0° deviation from prior information. The max error of heading is about 0.1°, standard deviation 0.05°; the max error of pitch is about 0.2°, standard deviation 0.08°.

In fact, prior information is not exactly correct and always has a measurement error. Baseline length error is very small and can sometimes be ignored, but angular error cannot be ignored for its inaccurate measurement of other navigation systems or sensors. In the computation of this proposed new method, we substitute angular prior information for the true value. It affects the ambiguity solution and decrease success rate. 

**Figure 3:**
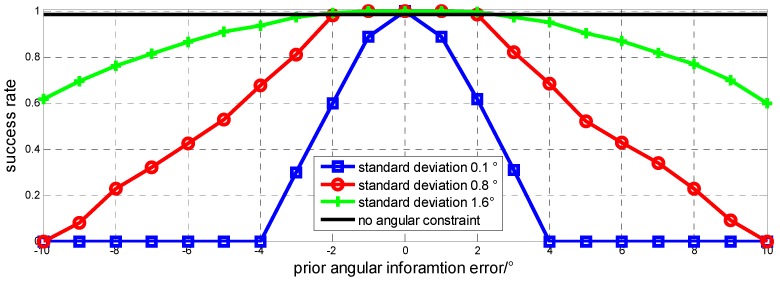
Prior angular information error affects success rate of ambiguity fix, heading as example.

In the first static experiment, the true value of the heading is known as 23.5°. In order to test how the angular prior information error affects ambiguity solution, we add some error to the true value and set its standard deviation as real measured prior information for simulation. As Figure 3 shows, with a given standard deviation, the bigger the error is the lower the success rate is. The proposed method is sensitive to the error when standard deviation becomes smaller. With small standard deviation, the success rate can achieve a very high level when angular prior information error is small, but will decrease rapidly when errors increase. On the contrary, when standard deviation is big, the new method does not become too sensitive to prior information error, but at the same time the performance of constraint becomes worse.

Figure 3 also shows that, set standard deviation of 0.8° as example, success rate decreases along with prior information error increases, but when error is within 2°, the new method still performs better than that without angular constraint. Pitch is similar to heading, no need to test again. 

According to the simulation result, we can reconstruct the objective function to achieve a better performance of baseline vector constraint. When prior information is not much reliable, we set a bigger standard deviation or even eliminate the relevant angular constraint items in objective function. When prior information is reliable with a higher precision, we set a smaller standard deviation to make a tight constraint. Using the inverse of variance of each item as weight value in Equation (5) is just in according with that idea, *i.e.*, smaller standard deviation means reliable prior information and thus has a bigger weight value.

### 4.2. Dynamic Test 

**Figure 4:**
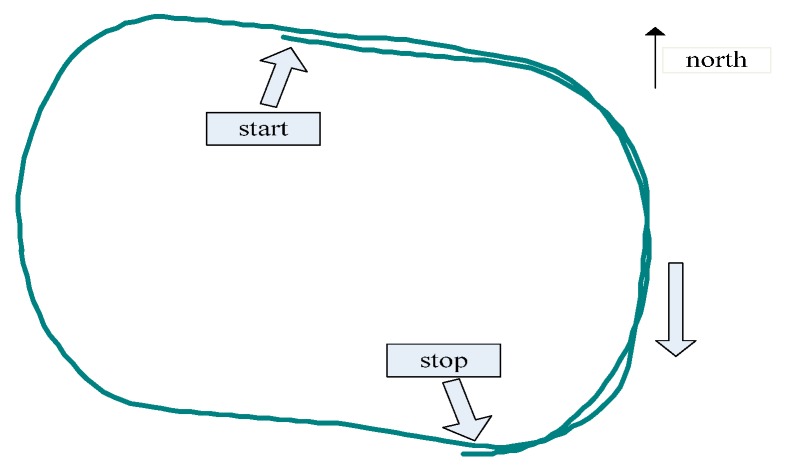
Vehicle move path.

**Figure 5:**
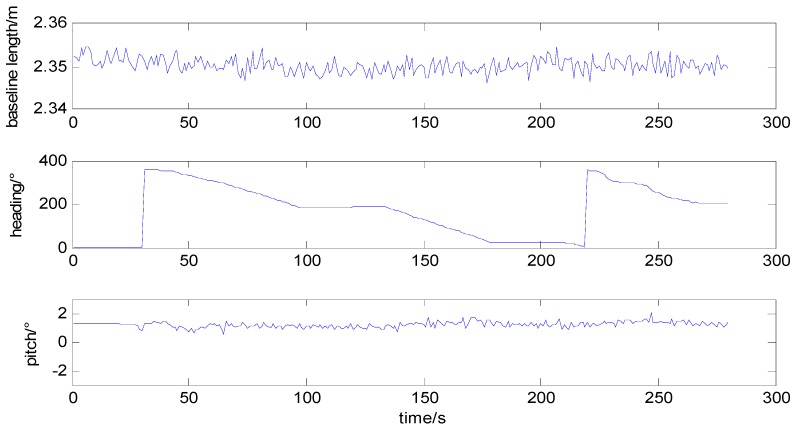
Heading and pitch determination during the test.

Fasten two antennas along the axis of experiment vehicle, drive it along 400 m sports track one lap and a half, and determine attitude continuously. Before it moves, prior baseline information is measured as length 2.353 m, heading 0.253°, pitch 1.837°, set the standard deviation of baseline, heading, and pitch are 0.5 mm, 0.8°, 0.6°, respectively. The vehicle movement path is as shown in Figure 4. GNSS determined baseline length and attitude are shown in Figure 5.

From Equation (6), heading is defined as the angle between east and the horizontal project of baseline, increases from zero to 360° counterclockwise. Pitch is defined as the angle between baseline and horizontal plane. During the first 30 s, the vehicle stands still, heading and pitch remain unchanged, the integer ambiguity is resolved by new method with single frequency, single epoch. Figure 5 shows that during the move, baseline length is measured as 2.35 m which corresponds to prior information; heading is changing corresponding to the vehicle moving track; pitch is about 1.3°, maximum floating value is about 1° corresponding to the flat track of ground. The vehicle movement track is obtained by reference antenna pseudorange positioning. The experiment result indicates that baseline length, heading, and pitch are all measured correctly, which is owing to the correctly fixed ambiguity.

## 5. Conclusions

This contribution utilizes baseline vector constraint, fully concerns the prior information of baseline length, heading, and pitch, integrates it into objective function. A fast and reliable new method is proposed to increase the success rate of single frequency, single epoch ambiguity fix.

1. Expand traditional baseline length constraint to baseline vector constraint of three-dimensions. The inverse of variance of each item in objective function is set as a weighted value. Compared to length constraint only, the angular constraint item can effectively avoid fixing the wrong ambiguity which results in attitude deviating from prior information to some extent. Thus, it can increase the success rate compared to only baseline length constraint.
2. Substitute ellipsoidal search space for non-ellipsoidal search space to make it suited for LAMBDA search process, and expand it gradually. An accelerating strategy is proposed to avoid calculating relevant nonlinear least square items in objective function repeatedly, which makes searching more efficient.
3. The performance of vector constraint depends on the accuracy of prior information. Generally, baseline is very precise, while angular information is not. When prior information is not reliable enough, ambiguity fix success rate decreases, but when angular error is within a given range (2° in simulation), it still performs better than the traditional method with only length constraint.

Different from traditional single frequency, single epoch attitude determination method, and this paper utilizes baseline vector prior information fully and rigorously. On the other hand, eliminating some extra wrong ambiguity candidate vectors by inequality is an effective method to accelerate search. Experiment results show that the proposed new method can achieve a very high ambiguity fix success rate up to 100%. We also test the affection by prior information error in simulation to prove its robust performance. A final dynamic vehicle experiment further verifies the reliability of this new method.
